# Anti-Inflammatory Role of the Klotho Protein and Relevance to Aging

**DOI:** 10.3390/cells13171413

**Published:** 2024-08-24

**Authors:** Gérald J. Prud’homme, Qinghua Wang

**Affiliations:** 1 Department of Laboratory Medicine and Pathobiology, University of Toronto, 220 Walmer Rd, Toronto, ON M5R 3R7, Canada; 2 Department of Laboratory Medicine, Keenan Research Centre for Biomedical Science, Unity Health Toronto, Toronto, ON M5B 1W8, Canada; 3 Department of Endocrinology and Metabolism, Huashan Hospital, Shanghai Medical School, Fudan University, Shanghai 200030, China; 4 Shanghai Innogen Pharmaceutical Co., Ltd., Shanghai 201318, China

**Keywords:** aging, Alzheimer, atherosclerosis, inflammasome, FGF23, fibrosis, Klotho, NF-κB, sarcopenia, TGF-β

## Abstract

The α-Klotho protein (hereafter Klotho) is an obligate coreceptor for fibroblast growth factor 23 (FGF23). It is produced in the kidneys, brain and other sites. Klotho insufficiency causes hyperphosphatemia and other anomalies. Importantly, it is associated with chronic pathologies (often age-related) that have an inflammatory component. This includes atherosclerosis, diabetes and Alzheimer’s disease. Its mode of action in these diseases is not well understood, but it inhibits or regulates multiple major pathways. Klotho has a membrane form and a soluble form (s-Klotho). Cytosolic Klotho is postulated but not well characterized. s-Klotho has endocrine properties that are incompletely elucidated. It binds to the FGF receptor 1c (FGFR1c) that is widely expressed (including endothelial cells). It also attaches to soluble FGF23, and FGF23/Klotho binds to FGFRs. Thus, s-Klotho might be a roaming FGF23 coreceptor, but it has other functions. Notably, Klotho (cell-bound or soluble) counteracts inflammation and appears to mitigate related aging (inflammaging). It inhibits NF-κB and the NLRP3 inflammasome. This inflammasome requires priming by NF-κB and produces active IL-1β, membrane pores and cell death (pyroptosis). In accord, Klotho countered inflammation and cell injury induced by toxins, damage-associated molecular patterns (DAMPs), cytokines, and reactive oxygen species (ROS). s-Klotho also blocks the TGF-β receptor and Wnt ligands, which lessens fibrotic disease. Low Klotho is associated with loss of muscle mass (sarcopenia), as occurs in aging and chronic diseases. s-Klotho counters the inhibitory effects of myostatin and TGF-β on muscle, reduces inflammation, and improves muscle repair following injury. The inhibition of TGF-β and other factors may also be protective in diabetic retinopathy and age-related macular degeneration (AMD). This review examines Klotho functions especially as related to inflammation and potential applications.

## 1. Introduction

The multifunctional α-Klotho protein (henceforth Klotho or KL) has major protective effects against aging and its associated pathologies [1,2,3,4,5,6,7,8]. These antiaging properties were first observed over 25 years ago in mice homozygous for a hypomorphic α-*Klotho* gene (*Kl^kl/kl^* phenotype) [9]. These mice had stunted growth, organ atrophy, hyperphosphatemia, hypercalcemia, marked calcification of arteries, cardiac hypertrophy, emphysema-like lung disease, osteopenia, sarcopenia, cognitive deficit, multi-organ fibrosis and a short lifespan. In contrast, transgenic mice overexpressing Klotho were healthy and had an increased lifespan [10].

**Figure 1:**
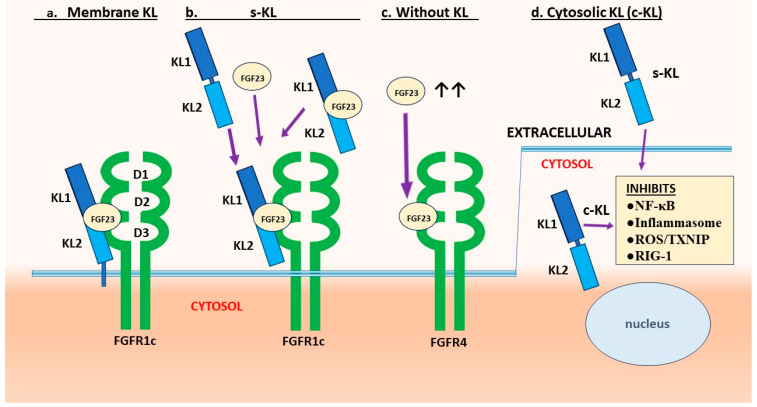
Klotho as a coreceptor for FGF23 and other functions. Membrane-bound Klotho has two extracellular domains (KL1 and KL2), a single-pass transmembrane segment (TM), and a short non-signaling cytoplasmic tail (CYT). The soluble form (s-Klotho) consists of the KL1 and KL2 domains only, and it is generated by cleavage on the surface of the cell. (**a**) Klotho (membrane-bound or soluble) binds to FGFR1c (or other FGFR) through an extension of the KL2 domain. FGF23 attaches at high affinity in a groove formed by segments of KL1, KL2 and the FGFR to induce FGFR signaling. (**b**) s-Klotho binds to an FGFR (most often FGFR-1c) and functions as a FGF23 coreceptor. FGF23 has a much higher affinity for s-Klotho than to FGFR and might associate with s-Klotho the circulation. (**c**) At very high concentrations (typically in renal failure), FGF23 binds directly to FGFR4 to induce signaling. (**d**) A cytosolic form of Klotho (denoted c-Klotho here) inhibits key intracellular functions (NF-κB activation and other). Extracellular s-Klotho can penetrate into the cell (unknown mechanism) and exert functions similar to c-Klotho. **Abbreviations:** ↑, increased; c-KL, cytosolic Klotho; D1, D2 and D3, extracellular domains of FGFRs; FGF23, fibroblast growth factor 23; FGFR, FGF receptor; KL, α-Klotho; ROS, reactive oxygen species; RIG-1, retinoic acid-inducible gene I; s-KL, soluble Klotho; TXNIP, thioredoxin-interacting protein.

**Figure 2:**
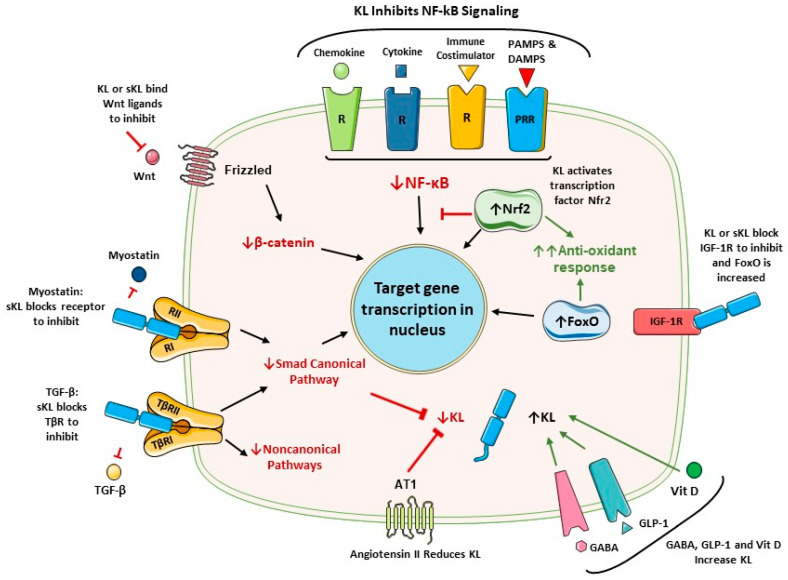
FGF23-independent functions of Klotho. Klotho binds to the TGF-β receptor and prevents TGF-β action. Klotho also binds to other TGF-β family receptors to suppress myostatin, and this increases muscle mass. Klotho blocks the NF-κB inflammatory pathway mainly by preventing nuclear translocation of the DNA-binding components. Klotho increases Nrf2, which counteracts NF-κB and induces multiple antioxidant enzymes. Klotho blocks IGF-1 receptor signaling. This activates FoxO and generates antioxidant responses. Klotho inhibits the Wnt pathway by blocking soluble Wnt ligands. Klotho expression is increased by the activation of either vitamin D, GLP-1 or GABA receptors. Klotho expression is suppressed by angiotensin II activation of the AT1 receptor. NF-κB activation and TGF-β/Smad signaling also suppress. There are other physiological regulators of Klotho production, as presented in Section 2.5. **Abbreviations:** AT1, angiotensin II type 1 receptor; Ang II, angiotensin II; DAMPs, damage-associated molecular patterns; FoxO, forkhead boxprotein O; GABA, γ-aminobutyric acid; GLP-1, glucagon-like peptide 1; IGF-1, insulin-like growth factor 1; IGFR, IGF-1 receptor; KL, Klotho; NF-κB, nuclear factor κB; Nrf2, nuclear factor-erythroid 2-related factor 2; PAMPs, pathogen-associated molecular patterns; R, receptor; sKL, soluble Klotho; TGF-β, transforming growth factor β; TβRI, TGF-β receptor type 1 (ALK5); TβRII, TGF-β receptor type 2; Vit D, vitamin D.

Subsequent studies revealed that the encoded Klotho protein exists as either a single-pass membrane protein or as a soluble form with endocrine properties (s-Klotho) [11,12,13,14]. It was established that Klotho is produced predominantly by renal tubular cells. It associates with some fibroblast growth factor (FGF) receptors (FGFRs) to form a high-affinity receptor for FGF23 (Figure 1a,b). Without Klotho, FGFRs have low affinity and are not activated, although as an exception, FGFR4 can respond to very high levels of FGF23 (Figure 1c). FGF23 is an endocrine FGF (eFGF) produced in bone by osteocytes and osteoblasts, which regulates phosphate/calcium homeostasis primarily by increasing the excretion of phosphate (phosphaturic effect) and reducing active vitamin D synthesis [1,15]. The FGF23 interaction remains the best characterized function of Klotho. Nonetheless, Klotho has other actions that appear independent of FGF23 (Figure 1d and Figure 2).

Klotho deficiency has been linked to numerous inflammatory and degenerative diseases involving the kidneys, cardiovascular system, brain and other organs [1,5,7,16]. Importantly, Klotho is thought to counteract chronic low-grade inflammation-related aging (inflammaging) and to extend the lifespan. Klotho levels inversely correlate with a systemic immune-inflammation index (SII) [17]. However, in most cases, the mode of action of Klotho is not well understood. The analysis of Klotho action is complicated by its interaction with numerous major pathways that affect practically all cellular functions. For instance, it inhibits NF-κB and TGF-β, which both generate multi-action signaling. These two pathways interact with each other and other pathways, including bone morphogenetic proteins (BMPs), Notch, Hedgehog, Hippo (TAZ/YAP), MAPK, PI3K/AKT and JAK/STAT [18,19,20,21]. In addition, Klotho inhibits insulin-like growth factor-1 (IGF-1) and Wnt, as reviewed [22,23,24].

Here, we focus primarily on the blockade of NF-κB and TGF-β as it relates to inflammation, fibrosis and other age-related disease. This involves the inhibition of the NLRP3 inflammasome, and a reduction in endoplasmic reticulum (ER) stress, reactive oxygen species (ROS) and tissue fibrosis. This is of major interest because Klotho levels can be enhanced clinically, and there is the possibility of therapeutic intervention.

## 2. Overview of Klotho Structure, Function and Regulation

### 2.1. Membrane-Bound and Soluble Klotho

The molecular structure of Klotho and its physiological action as a coreceptor for FGF23 have been extensively studied [6,15,16,25,26,27,28,29,30,31]. Klotho is a single-pass membrane protein with a short cytoplasmic segment (10 aa) [26,32]. The human form has 1012 aa (130 kD). The extracellular portion consists of two domains of similar size, which are designated KL1 and KL2. Circulating s-Klotho is generated by shedding. This is accomplished by extracellular proteases, mainly ADAM10 and ADAM17 (α-secretases), which cleave the ectodomain to release a KL1/KL2 soluble portion (s-Klotho) [32,33]. Smaller KL1 or KL2 fragments might also be generated, but this remains unclear. An alternative soluble KL1 form has been proposed, but it has premature termination codons and is degraded [34]. Crystal structure studies revealed how Klotho interacts with FGF receptors (often FGFR1c) to form a high-affinity receptor for FGF23 [26]. Heparan sulfate contributes to the formation of the signaling FGFR [35].

### 2.2. KL1 versus KL2 Domain Functions

Klotho can bind to FGFR 1c, 3c and 4 [26,36], but FGFR1c appears to be principal receptor on many cell types. Klotho binds to FGFR-1c through an extension of its KL2 domain, and FGF23 binds into a groove formed by segments of KL1, KL2 and the FGF receptor [26,35] (Figure 1a). Thus, KL2 is essential for receptor assembly and high-affinity FGF23 binding. In contrast, the KL1-domain or KL1-derived peptides mediate several of the functions attributed to s-Klotho (blockade of TGF-β, Wnt, and other), as presented in other sections. s-Klotho (consisting of both KL1 and KL2) can bind to FGFRs and act as a coreceptor for FGF23 [26,35] (Figure 1b). Thus, in tissues that lack Klotho but express an appropriate FGFR, circulating s-Klotho potentially acts as a roaming FGF23 coreceptor [6,37,38,39].

Notably, s-Klotho (KL1/KL2) can bind free FGF23 directly through two FGF23 C-terminal binding sites [28,31]. The affinity of FGF23 for Klotho (K_D_ = 14 nM) is three or four orders of magnitude greater than for FGFRs [28]. Therefore, circulating s-Klotho bound to FGF23 can form (Figure 1b) and potentially attach at high affinity to FGFRs [38,39]. The extent to which s-Klotho influences FGF23 signaling is not clear. At very high concentrations, FGF23 activates the FGFR4 receptor without Klotho (Figure 1c). This can occur in renal diseases, and it promotes cardiac pathologies [6,38,39,40,41]. Furthermore, s-Klotho regulates the function of some ion channels and transporters on the cell surface [31] by mechanisms that are not well understood.

### 2.3. Evidence of Cytosolic Klotho Action

Some activities of Klotho appear to be intracellular (Figure 1d). This form is denoted cytosolic (c-Klotho) here, but it is not well characterized. In accord, Klotho bound directly to a cytoplasmic inhibitor of κB (IκB), which is a component of the NF-κB pathway [42]. In the rat cardiac myoblast cell line H9c2 (2-1), intracellular Klotho suppressed apoptosis and colocalized with Hsp70 [43]. Others constructed a non-secreted KL1 segment (lacking a signal peptide) that interacted with and blocked RIG-1 [44], which is a sensor of RNA viruses. They showed by co-immunoprecipitation that intracellular KL1 directly bound to RIG-1. Nakayama et al. [45] fractionated HK-2 renal cells and identified Klotho in all cellular localizations, i.e., the membrane, cytoplasm, cytoskeleton and nuclear. Klotho protected chromosomal DNA against radiation damage. Others also report Klotho in the nucleus [46].

The addition of s-Klotho to the culture medium reversed the lack of intracellular Klotho [42,47]. In vivo, the injection of s-Klotho, KL1 or Klotho peptides blocked NF-κB activation, RIG-1 or other intracellular pathways [5,48,49,50]. This appears due to the endocytosis of Klotho, but the mechanism is unknown. In cells that are FGFR positive, endocytosis might occur after s-Klotho binds to that receptor. FGFR-mediated endocytosis has been reported for the closely related β-Klotho (a coreceptor for FGF19 and FGF21) [51]. FGFR1 is highly efficient at endocytosing ligands [52,53], and FGF21–FGFR1c–βKlotho complexes were internalized by clathrin-dependent endocytosis [51].

### 2.4. Organs and Cells That Produce Klotho

In addition to the kidney, Klotho is produced in the brain, endocrine glands (parathyroid, pancreatic β cells, other), blood vessels, skin, gastrointestinal epithelial cells, and immune cells [4,5,49,54,55]. Expression in blood vessels might be low or even negative [56]. However, several investigators have identified Klotho (including full-length) in the intima, media and endothelial cells of human arteries [54,57]. Furthermore, human endothelial cells express FGFR1 and respond to FGF23 [55,58]. Circulating Klotho might protect endothelial cells against injury. This is consistent with the results of parabiosis experiments [59,60].

### 2.5. Physiological Regulators of Klotho Expression

Klotho expression is upregulated or downregulated by several factors (Figure 2). In inflammatory disease, it is downregulated by inflammatory cytokines that activate NF-κB [32,61,62]. In contrast, PAX4 and PPARγ are factors that positively regulate expression [32], and this is relevant to diabetes. Vitamin D response elements in the promoter upregulate expression [32]. A neurotransmitter (GABA) and an incretin hormone (GLP-1) increase Klotho production in pancreatic β cells [5,47]. GABA also increases Klotho production in the kidneys [47].

Angiotensin II is a strong inhibitor of Klotho transcription [63]. Thus, drugs that inhibit the renin–angiotensin system (RAS) increase Klotho, as we previously reviewed [5]. Similarly, HMG-CoA reductase is inhibitory, and this can be antagonized by statin drugs [64,65]. Of interest, FGF23 also represses Klotho transcription [66]. In end-stage kidney disease, FGF23 levels can be increased by 1000-fold, whereas Klotho is reduced. Such excessive FGF23 levels are toxic to endothelial cells [67]. FGF23 overexpression accentuates systemic inflammation, multi-organ injury, fibrosis and cardiac pathologies (hypertrophy, cardiomyopathy and remodeling) [1,29,55,68]. TGF-β/Smad-signaling is another pathway that opposes Klotho expression [69]. Epigenetic regulation also occurs. For example, the epigenetic dysregulation of Klotho in renal disease includes promoter methylation, histone acetylation, the action of transcription factors (TF) and miRNAs [70].

## 3. Anti-Inflammatory Activities of Klotho

### 3.1. Klotho Inhibits NF-κB

Inflammation is an essential response to tissue injury, whether it is caused by infection, trauma, toxins, drugs, radiation, autoimmunity, or tissue necrosis. Chronic inflammation, often low-grade, plays a major role in age-related diseases, in a process termed inflammaging [71,72,73,74]. Inflammation can increase cell death, cellular senescence, telomere attrition, genomic instability, DNA damage, aberrant DNA methylation, impaired proteostasis and mitochondrial dysfunction [73]. It is a major cause of fibrosis in multiple organs. Klotho exerted potent anti-inflammatory activities in preclinical disease models. The mechanisms are not fully elucidated, but inhibition of the NF-κB pathway appears to be a key factor [4,5,49].

**Figure 3:**
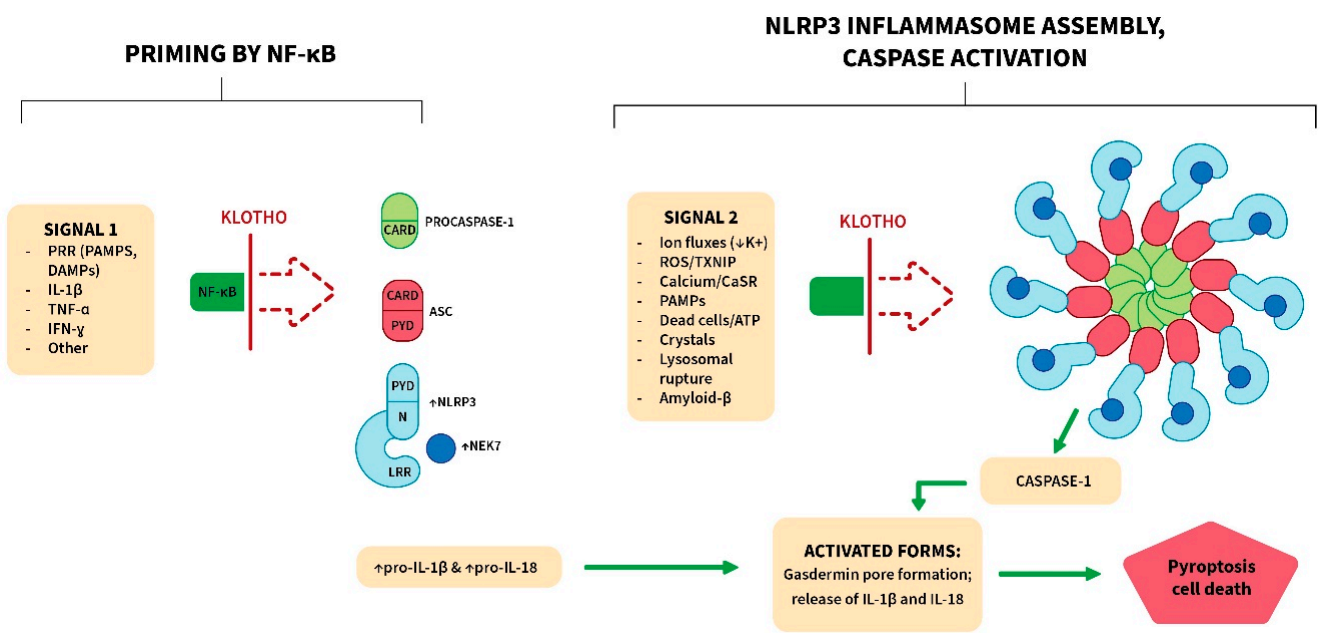
Klotho inhibits the priming (signal 1) and activation (signal 2) of the NLRP3 inflammasome. In resting cells, the levels of NLRP3, NEK7, pro-IL-1β and pro-IL-18 are low, but they are markedly increased by NF-κB signaling (priming; signal 1). This allows the assembly and function of the cytoplasmic NLRP3 inflammasome. Activation of this inflammasome can be induced by several events as listed (signal 2), although the mechanisms are not fully elucidated. The assembled NLRP3 inflammasome is a wheel-like structure with branches composed of a sensor (NLRP3), an adaptor protein (ASC) and an effector protein (procaspase-1). ASC links NLRP3 and procaspase-1 through PYD–PYD and CARD–CARD domain interactions. Once the inflammasome is fully activated, caspase-1 is generated, and it cleaves pro-IL-1β, pro-IL-18 and gasdermin D to generate their active forms. Gasdermin D forms pores in the membrane, allowing the release of IL-1β and IL-18 but also inducing cell death by pyroptosis. Final rupture of the cell membrane and cell lysis appear to depend on a ninjurin protein denoted NINJ1 [100]. Klotho inhibits signals 1 and 2, and it mitigates the inflammatory response (see Table 1). **Abbreviations: ↓,** decreased; ↑, increased; CARD, C-terminal caspase recruitment domain; CaSR, calcium sensing receptor; IFN-γ, interferon γ; NF-κB, nuclear factor κB; DAMPs, damage-associated molecular patterns; LRR, leucine-repeat rich; N, NATCH domain; NEK7, NIMA-related kinase 7; PAMPs, pathogen-associated molecular patterns; PRR, pattern-recognition receptor; PYD, pyrin domain; TNF-α, tumor necrosis factor α; TXNIP, thioredoxin-interacting protein.

The canonical p50/p65 heterodimer is the main NF-κB transcription factor in the majority of cell types. Canonical signaling is triggered by a wide variety of signals (Figure 3). This includes cytokines (e.g., IL-1β, TNF-α), chemokines, NOD-like receptors (NLR), Toll-like receptors (TLR), RIG-1-like receptors (RLR), stimulator of interferon genes (STING), receptor for advanced glycation end products (RAGE), lymphocytic receptors for antigen, costimulatory molecules, and other receptors [75,76,77]. NLRs and TLRs are pattern-recognition receptors (PRRs), which respond to diverse pathogen-associated molecular patterns (PAMPs) produced by infectious agents. NF-κB can also be activated by many self components. These can be products of dead cells, pathologic deposits (e.g., β-amyloid, urate crystals) and several other stimuli [78]. These are often termed damage-associated molecular patterns (DAMPs) and contribute to aseptic inflammation and age-related pathologies.

The suppression of NF-κB signaling by Klotho is well documented [42,47,48,79,80,81,82,83,84,85,86] (Table 1). Klotho inhibited NF-κB nuclear translocation in endothelial cells induced by TNF-α or circulating uremic toxins that create oxidative stress and cellular senescence (Table 1). The addition of s-Klotho to the culture medium attenuated the IFN-γ-induced nuclear translocation of NF-κB p65, indicating inhibition of the canonical pathway [5]. In murine cardiomyopathy, Klotho prevented the degradation of IκB (inhibitor of κB), preventing NF-κB p65 nuclear translocation, [82]. This was associated with nuclear factor-erythroid 2-related factor 2 (Nrf2) activation. In some studies, TLR4 was either degraded or inhibited [83,84,85]. TLR4 is a receptor for lipopolysaccharide (LPS) and several other ligands, which signals through NF-κB pathway, and it plays an important role in sepsis.

In human alveolar macrophages stimulated with cigarette smoke extract, Klotho bound directly to IκB and prevented its degradation, which blocked NF-κB signaling [42]. The knockdown of Klotho with siRNA increased NF-κB activation [42,47]. Adding s-Klotho to cultures reversed the effects of knockdown. NF-κB binds to the Klotho promoter and inhibits transcription [87] whereas Klotho suppresses NF-κB activation, suggesting competition between these events.

### 3.2. Klotho Inhibits the NLRP3 (NOD-Like Receptor Pyrin Domain Containing 3) Inflammasome

The inflammasomes are cytoplasmic molecular complexes that sense danger signals (infection, cell damage, tissue pathologies) and activate caspases (often caspase-1). These caspases activate pro-IL-1β and pro-IL-18 by cleavage. Caspase-mediated cleavage also activates gasdermin D, and it then forms pores in the membrane. The pores allow the release of mature IL-1β and IL-18, and they also induce a lytic form of cell death termed pyroptosis (Figure 3). There are multiple inflammasomes (e.g., AIM2, NLRP1, NLRP3, NLRC4, and PYRIN), each sensing a different set of danger signals [88,89,90]. Components of the different inflammasomes can interact together, and with other pathways, to induce cell death by pyroptosis, apoptosis and necroptosis, in a process termed PANoptosis [91,92]. Inflammasome formation occurs in immune cells and many other cell types. It is initiated by the activation of PRRs expressed on the cell membrane or in the cytoplasm [93]. Dysregulated inflammasome activation is the basis of genetic acute inflammatory syndromes (termed autoinflammatory), such as cryopyrin-associated periodic syndrome (CAPS) [88,94,95].

The NLRP3 inflammasome is the most extensively studied, and it responds to a much wider variety of stimuli than other inflammasomes [88,89,90]. For instance, it is activated in radiation-induced injury due to ROS generation and other events [96]. This leads to inflammation, pyroptosis, increased TGF-β action and subsequent tissue fibrosis. It plays a major role in infectious diseases where it generates acute inflammation and in gout in response to urate crystal deposition [97]. It has also been linked to chronic inflammatory conditions, including atherosclerosis and Alzheimer’s disease [96,98]. The key components of the NLRP3 inflammasome and its assembled form are depicted in Figure 3 [97,98,99,100,101,102,103]. As a late step, NEK7 binds to the LRR segment of NLRP3, and this promotes activation. Full activation of the NLRP3 inflammasome requires two signals [102] (Figure 3). Signal 1 is a priming signal delivered by NF-κB. In the cytosol of resting cells, NLRP3, NEK7, pro-IL-1β and pro-IL-18 are present in low amounts [102,103,104]. However, NF-κB activation increases these proteins, whereupon they can generate a functional inflammasome. Signal 2 (activation) is mediated by a variety of stimuli (Figure 3), but the mechanisms are not fully understood [105].

Klotho blocks signal 1 and counteracts events that generate signal 2 (Figure 3; Table 1). For instance, it inhibits ROS generation (signal 2 stimulator) by increasing Nrf2 action [106]. Sirtuin-1 (SIRT1) is also increased and appears to promote this antioxidant action [106]. The reduction in ROS prevents TXNIP action, and this obstructs signal 2. Klotho also reduces calcium and phosphate levels, the generation of calciprotein particles (CPPs) and calcium deposition in tissues. This mitigates the activation of the inflammasome by the calcium-sensing receptor (CaSR). In addition, Klotho protects against cell death. We hypothesize this prevents the release of inflammasome-activating molecules from dead cells, although the contribution of this factor is not known.

Romero et al. [107] showed that Klotho inhibits the NLRP3 inflammasome in endothelial cells. They reported that secreted IL-1β (produced by the inflammasome) can bind to the IL-1 receptor of human endothelial cells to amplify NF-κB and NLRP3 inflammasome activation in a positive feedback loop (autoactivation). Klotho blocked this vicious circle and mitigated endothelial dysfunction. In mice, in vivo treatment with soluble Klotho blocked IL-1β-mediated activation of the inflammasome. Klotho is also inhibitory in various preclinical disease models as shown in Table 1, including cardiomyopathy, diabetes, renal disease and neurodegenerative conditions [106,107,108,109,110,111,112,113,114,115,116,117].

**Table 1:** Klotho inhibits NF-κB and the NLRP3 inflammasome.

Klotho Treatment In Vivo and/or In Vitro	Mechanisms or Concurrent Events	Organ or Cells (Disease)	Reference
**NF-κB ↓**	TLR4 ↓	heart (aging)	[85]
		heart (cardiotoxicity)	[84]
		intervertebral disk (degeneration)	[83]
	Nrf2 ↑	heart (cardiomyopathy)	[82]
		kidney (diabetes)	[80]
	Blocked NF-κB p65 nuclear translocation	endothelial cells (+TNFα)	[79]
		endothelial cells (+uremic toxins)	[48]
		pancreatic β cells (in culture)	[47]
		alveolar mϕ (cigarette smoke ext.)	[42]
		glomerular cells (+IFNγ; AG)	[86]
**NLRP3 Inflammasome ↓**	M2 microglial differentiation ↑, Aβ ↓	brain (Alzheimer-like disease mutant mouse)	[80]
	TXNIP ↓	brain, choroid plexus	[109]
		brain (neuroinflammation)	[113]
		heart (cardiomyopathy)	[111]
		cartilage (osteoarthritis)	[110]
	Nrf2 ↑	brain (temporal lobe epilepsy)	[115]
	NF-κB ↓	kidney (diabetes)	[114]
		testes (dioxin exposure)	[108]
		kidney (contrast-induced injury)	[116]
	NF-κB ↓ Blocked IL-1β auto-stimulation loop	endothelial-cell dysfunction	[107]
	neuronal pyroptosis ↓	brain (ischemic tolerance model)	[117]
	Sirtuin-1 ↑ Nrf2 ↑ apoptosis ↓ IL-1β ↓ TNFα ↓ IL-6 ↓	A549 human cell line (LPS-treated in vitro)	[106]

**Abbreviations:** ↓, decreased; ↑, increased; +, added to the culture; Aβ, amyloid-β; AG, Aicardi–Goutiere syndrome; ext., extract; IFNγ, interferon γ; LPS, lipopolysaccharide; Nrf2, nuclear factor-erythroid 2-related factor 2; mϕ, macrophage; TLR4, toll-like receptor 4; TNFα, tumor necrosis factor α; TXNIP, thioredoxin-interacting protein.

Several new drugs targeting inflammasomes are being investigated [118,119]. Colchicine and tranilast are old drugs known to block the NLRP3 inflammasome. Colchicine reduced the progression of atherosclerosis and cardiovascular disease in clinical trials [120,121]. This is a strong indication that inflammation contributes to atherosclerosis, but colchicine can be toxic and has a narrow therapeutic range. Tranilast has been applied for decades for the treatment of allergy, asthma and fibrotic diseases, mainly in Japan. It blocks the NLRP3 inflammasome directly [122] and has both anti-inflammatory and anti-cancer activities [123,124,125,126]. In preclinical work, Klotho appears to have fewer adverse effects than existing drugs while having a broader anti-inflammatory profile, but this requires further investigation.

## 4. Endoplasmic Reticulum (ER) Stress and Oxidative Stress

ER stress and oxidative stress are two major factors that lead to cellular senescence and cell death in aging and several diseases [71,73,74]. These cellular stress factors are notably prominent in inflammaging [74], and Klotho exerts protective effects as described below.

### 4.1. ER Stress

The ER is involved in the synthesis and folding of secreted and membrane-bound proteins, calcium storage and lipid synthesis [127,128]. It repairs or eliminates unfolded or misfolded proteins. Proteins that cannot be repaired are eliminated by either the ubiquitin–proteasome or the lysosome–autophagy systems. However, when these mechanisms are overwhelmed, unfolded or misfolded proteins accumulate, causing a condition termed ER stress. This activates the unfolded protein response (UPR).

The UPR is regulated by three major ER sensors, and under ER stress conditions, they are activated. Their combined action increases protein degradation and decreases protein synthesis. If ER stress persists, the cells undergo death by apoptosis. The UPR reaction associates with oxidative stress, autophagy and mitochondrial dysfunction. Banerjee et al. [129] reported that Klotho has a major role in suppressing ER stress. They found that the loss of Klotho could be linked to ER stress-induced apoptosis. Similarly, others have reported Klotho’s beneficial effects against ER stress [130,131,132]. However, the mechanisms involved remain unclear.

### 4.2. Oxidative Stress and Klotho’s Antioxidant Functions

ROS are generated during normal cellular functions. They are mainly produced by mitochondria and NADPH oxidase (NOX). ROS are eliminated by antioxidant enzymes, such as superoxide dismutase (SOD) and catalase. ROS overproduction (oxidative stress) damages several molecular constituents and can induce cell death [133]. Importantly, as outlined in other sections, ROS (along with other factors) can activate NF-κB and the NLRP3 inflammasome.

Klotho activates two major antioxidant pathways: Nrf2 and FoxO (Figure 2). Klotho was found to activate Nrf2 in preclinical disease models involving the kidneys, cardiovascular system, and the brain [115,134,135,136]. Nrf2 and the inflammatory NF-κB pathway are mutually inhibitory [137]. In accord, a large number of anti-inflammatory agents can activate the Nrf2 pathway by interfering with NF-κB activation [138,139,140,141].

The second major antioxidant mechanism enhanced by Klotho is mediated by the FoxO proteins (FOXOs), which are a subfamily of forkhead transcription factors [142]. In this case, Klotho blocks the IGF-1 receptor, which reverses the IGF-1-mediated inhibition of FOXO expression. This allows the FOXOs to migrate into the nucleus and protect against oxidative stress through the expression of enzymes such as catalase and manganese SOD [133]. For example, Klotho reduced damage in rat hearts with ischemia–reperfusion injury (IRI) [143]. These authors concluded that reduced oxidative stress and IRI was due to inhibition of the IGF1R/PI3K/AKT pathway.

## 5. Early Vascular Aging

A major deleterious aspect of aging is vascular disease. Pathologic changes appear gradually, especially vascular calcification and atherosclerosis. Early (premature) vascular aging (EVA) occurs in subjects with hypertension, diabetes, exposure to cigarette smoke and advanced kidney disease. Vascular calcification is a prominent feature of Klotho deficiency [144]. In *kl*/*kl* mice, these vascular changes are mitigated by a low-phosphate diet [1,145]. Calcium in the vessel wall can activate the CaSR [146], which encourages macropinocytosis, CPP (calciprotein particle) uptake, and activation of the NLRP3 inflammasome. 

The majority of individuals over 70 years old have vascular calcification in multiple vessels [147]. In one pattern, calcium is deposited extensively in the media of arteries (Monckeberg’s medial calcific sclerosis) [148]. Clinically, it often associates with systolic hypertension and other cardiovascular anomalies [147,149]. In most cases of vascular calcification, however, calcium deposition occurs in atherosclerotic lesions. Calcification also frequently involves heart valves, especially the aortic valve (calcific aortic stenosis).

Atheroslerosis is a very common and potentially lethal arterial disease. It can affect the aorta and all its major branches. In the coronary arteries, it can lead to thrombosis, obstruction and myocardial infarction (MI). Similar lesions occur in the brain and other organs. Atherosclerosis has traditionally been linked to hyperlipidemia, but the additional contribution of inflammation is now well recognized [94]. The atherosclerotic plaque frequently occurs in areas of endothelial stress or injury. Cholesterol accumulates in the core of the plaque and forms crystals, which can be phagocytosed by macrophages. This results in lysosomal disruption with the release of cathepsin B and subsequent activation of the NLRP3 inflammasome. Furthermore, ROS, TXNIP and calcium aggregates can all contribute to inflammasome activation. This releases IL-1β and stimulates further infiltration of the plaque by inflammatory cells. Damage to endothelial cells, erosion, or plaque rupture lead to the adherence of platelets and formation of a thrombus, which often blocks the lumen completely (as in coronary thrombosis) [150].

In clinical studies, Klotho deficiency has been associated with a high risk of atherosclerotic disease [2,6,151]. Indeed, low levels appear to be an early marker of this disease. Low Klotho may also contribute to cardiac microvascular disease [152]. Klotho inhibits several factors that contribute to atherosclerosis. It limits inflammatory factors, such as inflammasome activation and pyroptosis, which drive this disease process [107,153,154]. It ameliorates medial calcification, endothelial injury/dysfunction and hypertension, and generally counters EVA [2,6,57,151,155,156]. In rodents, Klotho reduces vascular calcification, but there have been few studies of atherosclerosis. Yang et al. [81] found that Klotho treatment alleviated platelet-dependent atherosclerotic lesions in *apoE^−/−^* mice (with or without chronic kidney disease). Interestingly, treatment was associated with reduced platelet activation and less thrombosis.

## 6. Blockade of TGF-β Cytokine Family Members

### 6.1. Signaling TGF-β Receptors Are Blocked

The ability of Klotho to inhibit TGF-β is well established. The TGF-β family consists of at least 33 members. TGF-β is by far the best studied. It is a pleiotropic cytokine produced by many cell types and found in the circulation. Almost all cell types express receptors. Klotho was initially found to block by binding to the TGF-β type 2 receptor (TβRII) [157]. However, more recently, it has been reported to also bind to the type 1 receptor (TβRI, or ALK5) as well as to the receptors of some other TGF-β family members (myostatin, GDF11 and activins) [158]. TGF-β and cytokines of this group have wide-ranging activities on disease and aging as related, for example, to stem cell senescence, organ fibrosis, cardiovascular disease, immune dysfunction, muscle atrophy and cancer [123,159,160,161,162,163]. 

### 6.2. Klotho Inhibits Epithelial-Mesenchymal Transition (EMT) and Fibrosis

EMT is a process that occurs during embryogenesis and also in adult life as part of healing and various pathologies [164]. In this process, epithelial cells acquire mesenchymal markers (e.g., vimentin, collagen-1 and N-cadherin) and lose epithelial markers (e.g., cytokeratins, E-cadherin and Epcam) [164,165]. EMT is often a precursor to fibrosis.

TGF-β is the most potent stimulator of EMT, but other mediators contribute. Thus, TGF-β, Wnt, Hedgehog, hepatocyte growth factor (HGF), platelet-derived growth factors (PDGF) and neuropilins are all involved [160,166]. TGF-β/Smad signaling upregulates EMT-associated transcription factors such as Snail1, Twist, β-catenin and Zeb1/2, which drive the expression of several EMT-associated genes [164,165]. In tumors, EMT is thought to promote invasiveness and metastasis. Moreover, EMT has been associated with the acquisition of a cancer stem cell (CSC) phenotype [124,167]. The CSCs have a high metastatic potential and are markedly chemoresistant. This contributes to cancer recurrence and progression.

Klotho blocks both TGF-β and Wnt/β-catenin to prevent EMT and fibrosis. For instance, Klotho inhibited EMT and fibrosis in diabetic renal disease [168] and in a rat renal transplant model [169]. Similarly, it reduced EMT in TGF-β2-induced retinal lesions [170]. High glucose levels inhibit Klotho expression, at least in some cell types [163]. In human mesangial cells treated with high glucose, Klotho was depressed, and extracellular matrix (ECM) protein production was enhanced. This was reversed by the forced expression of Klotho [163], which inhibited TGF-β1/Smad3 signaling. Klotho blocked TGF-β1 and suppressed renal fibrosis and cancer metastasis in mice [157]. It also reduced fibrosis in a model of cardiomyopathy [13].

A 30 amino acid peptide (KP1, from the KL1 domain) blocked the TGF-β receptor and prevented renal fibrosis in mice [171]. A different Klotho KL1 domain peptide of similar size (P6) blocked Wnt1 and Wnt3a, and it prevented fibrosis and other lesions in mouse diabetic kidney disease [172]. The mechanisms involved in anti-cancer activities might differ from fibrosis. Abboud et al. [173] reported that a longer sequence of KL1 (KL 1-340) was required to inhibit Wnt3a expression and colony formation in cancer cells. Overall, these findings confirm that Klotho, or Klotho-derived peptides, can counteract EMT and fibrosis.

### 6.3. Endothelial–Mesenchymal Transition (EndMT)

The role of inflammasome activation in atherosclerosis has been emphasized. However, EndMT is another process that is thought to promote the development of atherosclerotic plaques [149,174,175]. In EndMT, endothelial cells are induced to differentiate into mesenchymal cells, primarily under the influence of TGF-β, Wnt and Notch [160,174]. Under normal conditions, endothelial cells express quite low levels of TβR. This is dependent on ongoing signaling from FGFR1, which downregulates TβR. However, a decline of FGFR1 upregulates TβR expression and sensitizes the cells to respond to TGF-β [174,175,176]. Inflammation and related inflammatory cytokines severely downregulate FGFR1 expression and upregulate TβR, thus promoting EndMT. Klotho mitigated EndMT in unilateral ureteral obstruction by blocking TGF-β1 [177]. It also suppressed EndMT in the aorta of mice with chronic kidney disease [178].

### 6.4. Inhibition of Myostatin and Treatment of Sarcopenia

Klotho-deficient mice (*kl*/*kl*) have marked muscle atrophy (sarcopenia) [145]. There could be several factors involved, but the dysregulation of TGF-β, myostatin (TGF-β family) and other TGF-β family members appears to be important. Myostatin is a negative regulator of muscle mass [179,180]. It suppresses muscle differentiation and regeneration, and it promotes muscle atrophy as in sarcopenia. It has been linked to senile muscle atrophy and cachexia. Myostatin signals through type 2 receptors ActRII or ActRIIB, and type 1 receptors ALK-4 (ActRIB) or ALK5 (TβRI), and it employs the Smad2/Smad3 signaling pathway [181]. Thus, it shares key signaling elements with TGF-β and several other members of the TGF-β family. Interestingly, myostatin is closely related with growth and differentiation factor 11 (GDF11), which is another negative regulator of muscle mass [181]. Activins, which share receptors with myostatin and GDF11, similarly reduce muscle mass. The recent findings of Ohsawa et al. [158] suggest that Klotho blocks members of the TGF-β family that suppress muscle mass, including TGF-β1, myostatin, GDF11 and activins. Klotho blocked by binding to myostatin as well as to type I and type II serine/threonine kinase receptors of the TGF-β receptor family. Interestingly, the oral administration of a small drug antagonist for type I receptors (ALK4 and ALK5) reversed muscle atrophy and weakness in Klotho-deficient mice as well as in old wild-type mice. The authors postulated that s-Klotho counteracts TGF-β-induced sarcopenia. However, other factors are also involved in sarcopenia [182,183]. Inflammation contributes to the development of sarcopenia in cancer, chronic diseases and old age. In chronic renal failure, excess FGF23 and low Klotho promote inflammation and muscle wasting, and Klotho therapy is protective [184].

Klotho may have a physiological role in maintaining muscle mass in humans. For example, Klotho levels were inversely associated with low muscle mass in middle-aged subjects [182]. However, mechanisms have not been established. Sahu et al. [183] linked Klotho to muscle regeneration following cardiotoxin injury in mice. A genetic reduction of Klotho impairs muscle regeneration. They report that adult skeletal muscle expresses very low or undetectable levels of Klotho. Expression increases following injury in young mice, but much less in old mice. Nevertheless, Klotho improved muscle stem (satellite) cell (MuSCs) lineage progression and myofiber regeneration in aged muscle. It ameliorated mitochondrial DNA damage, ultrastructure and function. Importantly, systemic s-Klotho therapy was effective in these functions. Overall, the regulation of muscle mass is complex and differs in various conditions. The contribution of Klotho appears to be important but needs further investigation.

## 7. Role of Klotho in Diabetes

### 7.1. Protection against β-Cell Injury

Klotho is expressed in pancreatic β cells, and it is reduced in diabetic subjects [185]. Similarly, circulating levels of s-Klotho are frequently depressed in both type 1 diabetes (T1D) and type 2 diabetes (T2D) [186,187,188,189,190,191,192]. This is also observed in mouse models of the disease, such as db/db mice (T2D) [193] and NOD mice (T1D) [194]. Klotho-deficient (*kl*/*kl*; or knockout) mice have atrophic islets and low insulin levels [195].

Klotho therapy by gene transfer ameliorated disease in both T1D and T2D mice [185,196]. Similarly, Klotho protein therapy was effective in autoimmune NOD mice [194]. In NOD mice, insulitis was suppressed and β-cell mass was ameliorated. In vitro treatment of human β cells with Klotho increased insulin secretion and cell proliferation while reducing cell death [47]. A key finding was the ability of Klotho to suppress NF-κB activation. Furthermore, similar effects on β cells were observed when agents that increase Klotho expression were applied, such as GABA or a GLP-1 mimetic drug [5,47,197,198,199]. Importantly, in mice carrying xenotransplanted human islets, these β-cell protective effects were observed in vivo [200]. 

Importantly, FGFR1c is expressed by β cells [201]. Because Klotho and KLB (β-Klotho) are both expressed by β cells, it is likely these cells respond to FGF23 (dependent on α-Klotho) and FGF21 (dependent on β-Klotho). Furthermore, s-Klotho might act as a circulating coreceptor to increase FGF23 responses.

In both types of diabetes, several cell-death mechanisms have been proposed, including apoptosis, necroptosis, pyroptosis, ferroptosis and autophagy [202,203]. Glucotoxicity and inflammation are major pathogenic factors [203], and high glucose levels suppress Klotho expression. A common factor appears to be mitochondrial dysfunction and the excessive production of ROS. In β cells and other cells, oxidative stress induces the release of TXNIP (thioredoxin interacting protein) from the nucleus, which blocks the antioxidative action of thioredoxin and increases ROS [204,205]. ROS can activate NF-κB, and TXNIP is an activator of the NLRP3 inflammasome. Klotho may mitigate several of these deleterious factors, especially oxidative stress as well as the activation of NF-κB and the NLRP3 inflammasome.

### 7.2. Advanced Glycation End Products

The non-enzymatic glycation of proteins and other substrates is a natural feature of aging; however, in diabetic subjects, it is markedly accelerated by hyperglycemia. Chemically, reducing sugars react non-enzymatically with amino groups present on proteins and other molecules. This results in the glycation, damage and cross-linking of proteins, forming persistent advanced glycation end products (AGEs) [76,206,207]. AGEs are produced endogenously, but they are also present in many food items and cigarette smoke [76,208]. Over time, the AGEs deposit in basement membranes and many other sites, impairing function. They can inactivate enzymes. The glomerular basement membrane is a prominent target, and this leads to increased permeability and, at advanced stages, the nephrotic syndrome. AGEs deposit in blood vessels, accelerating atherosclerosis and microvascular pathology.

AGEs can bind to some PRRs. The most important is the receptor for advanced glycation end products (RAGE). This receptor binds many ligands including AGEs, β-amyloid, S100/calcineurin, LPS and nucleic acids [207]. After binding its ligands, RAGE stimulates a broad array of inflammatory pathways, including NF-κB, the NLRP3 inflammasome and the secretion of inflammatory cytokines [209,210,211]. It augments TGF-β production and Wnt activation. Of interest, exposure to oxidized protein products activated the Wnt/β-catenin pathway and injured glomerular podocytes [212]. In a soluble form, Klotho antagonized this RAGE-induced injury. Others reported that increasing AGE levels lowered Klotho production by human renal tubular cells (HK-2), and this was reversed by a phytochemical [213]. These studies suggest that Klotho can interrupt at least some RAGE-mediated deleterious actions.

### 7.3. Diabetic Retinopathy and Age-Related Macular Degeneration

Klotho has therapeutic potential against diabetic retinopathy (DR), as recently reviewed [214]. Diabetes accelerates atherosclerosis in medium and large vessels. Frequently, there is also microvascular disease involving small arteries and arterioles (arteriolosclerosis) as well as capillaries. Diabetic microangiopathy is prominent in the retina, peripheral nerves and kidneys. In DR, there is retinal capillary microaneurysm formation, exudates and macular edema; and at advanced stages, there is angiogenesis, hemorrhage, fibrosis and other lesions (proliferative DR). This can lead to blindness. Klotho is produced in the retina and appears essential to sustain normal retinal function [215]. Klotho might protect against DR by suppressing TGF-β signaling, epithelial-to-mesenchymal transition (EMT), ROS generation, vascular endothelial growth factor (VEGF) secretion and apoptosis [8,214]. However, its effectiveness as a treatment for human DR remains to be determined.

Klotho may also play a role in age-related macular degeneration (AMD). TGF-β2 (the major TGF-β form produced in the eye) is thought to be a major pathogenic factor in AMD [170]. Increases in vitreous TGF-β2 are associated with retinal fibrosis. Intravitreal injection of Klotho in mice reduced TGF-β2-mediated EMT as well as degenerative changes of the retinal epithelial cells [170]. This appears to be due to the blockade of the TGF-β receptor by Klotho. Klotho reduced retinal pigment cell senescence, EMT, as well as cytosolic and mitochondrial oxidative stress. Thus, Klotho has potential as a retinal disease therapeutic agent.

## 8. Protection against Neurodegenerative Pathologies

### 8.1. Klotho Production in the Brain

Klotho has a prominent protective role against neurodegenerative diseases, especially Alzheimer’s disease. Klotho exerts neuroprotective effects and/or improves cognition in humans and primates (Table 2) [7,216,217,218,219,220,221,222,223,224,225,226,227,228]. Its protective actions appear to extend beyond Alzheimer’s disease and include other forms of dementia, Parkinson’s disease and multiple sclerosis (MS).

Klotho is produced by the choroid plexus and present in the CSF. Importantly, it is expressed by both neurons and glial cells. It is present in gray matter areas and, notably, the hippocampus [131,229,230]. Klotho CSF levels and tissue expression in the brain decline with aging and early Alzheimer’s disease [231].

### 8.2. Neuropathologic Findings and the Role of Neuroinflammation

Alzheimer’s disease is the most common form of dementia. Specific mutations have been identified in the inherited forms, but most cases are sporadic and the cause is not known. Two pathologic features have attracted a great deal of attention. These are excessive numbers of Aβ (amyloid-β) plaques in the brain and the formation of neurofibrillary tangles (NFTs) [232]. The NFTs consist of aggregates of hyperphosphorylated tau proteins. They form inside neurons but can persist extracellularly when these cells die. Both Aβ and NFTs appear to be toxic to neurons and to play an important role in the pathogenesis of this disease. For instance, the administration of anti-Aβ monoclonal antibodies appears to produce some therapeutic benefit in early Alzheimer’s disease, albeit with adverse effects [233,234].

The most damaging event is excessive neuronal cell death. With aging, a gradual loss of some neurons is normal, but in Alzheimer’s disease, it is severe. Cell death can occur by apoptosis, necroptosis, pyroptosis, ferroptosis and probably other mechanisms [131,205]. Factors such as DNA damage, mitochondrial dysfunction, ROS production, ER stress, defective autophagy, NF-κB activation and inflammasome action can all occur and are likely subject to inhibition by Klotho.

Cognitive decline is an important aspect of inflammaging [113]. Indeed, neuroinflammation, inflammasome activation and associated pyroptosis are considered major pathogenic factors [235,236,237,238,239,240,241]. Importantly, Aβ can activate the NLRP3 inflammasome in microglia, neurons and other cells. Tau protein can also activate the NLRP3 inflammasome [242]. Other inflammasomes participate in this complex inflammatory environment (e.g., NLRP1, NLRP2, NLRC4). PRRs in the brain are numerous and include TLRs, NLRs, CaSR, RAGE, various scavenger receptors and other receptors [243,244]. TLR4 binds Aβ and is a major PRR for inflammasome activation in Alzheimer’s disease [205,245]. Most likely, inflammasome activation can also be either initiated or amplified by CaSR and RAGE, which are abundant in the brain. These receptors are polyspecific and can bind Aβ and several other ligands [243,246]. In diabetic subjects (especially T2D), there is a marked increase in the incidence of Alzheimer’s disease [247]. This is possibly related to the accumulation of advanced glycation end products in the brain and cerebral vasculature, which activate RAGE and consequently the NLRP3 inflammasome. Aβ can form an amyloid in medium and small cerebral arteries (cerebral amyloid angiopathy). This type of vasculopathy can lead to widespread microbleeds in the brain and associated injury. In sum, vascular disease can contribute to the onset of Alzheimer’s. Thus, diabetes, vascular dementia and Alzheimer’s disease can coexist.

### 8.3. Evidence That Klotho Counters Neurodegenerative Diseases

There is considerable evidence of Klotho-mediated neuroprotection in rodents as reported (Table 3) [60,80,109,115,131,248,249,250,251,252]. It was known from early reports that Klotho-deficient mice have severe cognitive dysfunction. In contrast, the overexpression of Klotho improved cognition. Subsequent studies showed that Klotho administration or genetic overexpression protects against Alzheimer-like disease in mouse models of this disease. It reduced neuroinflammation and ameliorated cognitive responses. Notably, it suppressed ROS, TXNIP, NF-κB activation, NLRP3 inflammasome action, and neuronal cell death (Table 1 and Table 3).

The hippocampus is one of the few sites where neurogenesis occurs in the brain of adults [131]. Hippocampal neurons play a critically important role in generating memory, and excessive loss of these neurons is a feature of Alzheimer’s disease. Klotho exerts potent protective effects on hippocampal neurons and appears to promote synaptic plasticity and neurogenesis [218,251,254]. It also enhances oligodendrocyte maturation and myelination, which might ameliorate demyelinating diseases such as multiple sclerosis [248].

Clinical trials on this topic have been often been restricted to the analysis of s-Klotho levels in the serum and the CSF (Table 2). These levels have been correlated to the scores obtained in various cognitive tests. In some cases, the diagnosis of neurodegenerative disease was supported by imaging analysis of Aβ deposition, biomarkers such as the levels of Aβ peptides and tau protein in the CSF, and other methods. Repeatedly, Klotho levels have been positively correlated with cognitive function. Low levels are associated with neurodegenerative disease [224,226]. The KL-VS variant of Klotho appears to have some protective effects although only in the heterozygous state and in some subgroups of the disease. It associates with higher levels of circulating Klotho and reduced phosphorylated tau (p-tau) protein in the CSF [220,222]. However, the mechanisms involved are not clear. The correlation between the serum and CSF levels of Klotho has not been consistent between studies, ranging from none, weak or high. Not surprisingly, CSF levels have been more reliable than serum levels in terms of an association with Alzheimer’s disease.

More direct evidence of Klotho action comes from the culture of human cerebral organoids, where the addition of Klotho to cultures reduced neuronal senescence [221]. Likewise, in cultures of human neural hippocampal precursor cells, it increased neuronal differentiation and reduced apoptosis [218]. Importantly, in nonhuman primates, Klotho protein injections improved performance in cognitive tests [225].

## 9. Therapy with Klotho

### 9.1. Klotho-Enhancing Strategies

**Figure 4:**
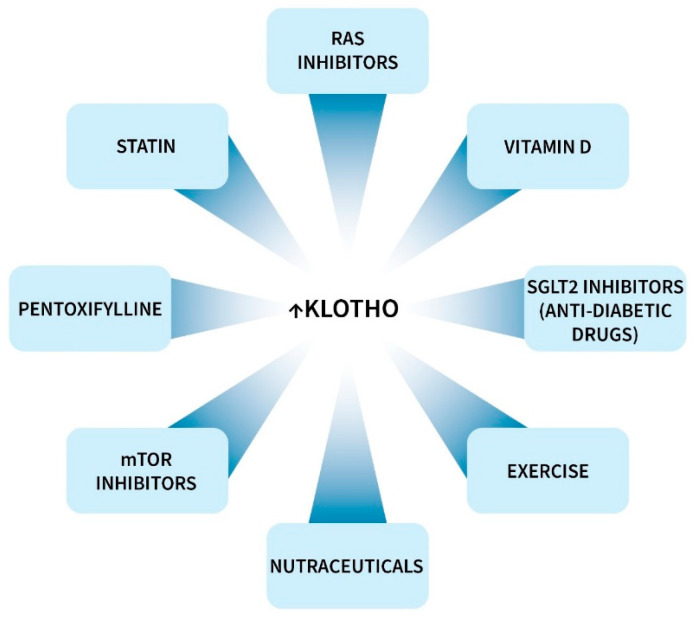
Drugs and factors that increased Klotho in clinical trials. Numerous agents increased Klotho in rodents and other species, but those listed in this figure increased Klotho in humans [5,7,8,255,256]. **Abbreviations:** mTOR, mechanistic target of rapamycin; RAS, renin–angiotensin system; SGLT2, sodium–glucose cotransporter.

Klotho protein or peptide injection is protective in rodents [5,7,255], but to our knowledge, clinical data are not available. Some clinical drugs, vitamins, nutraceuticals, traditional medicines and diet components increase circulating or tissue levels of Klotho, as recently reviewed by us and others [5,7,8,255]. Most of this work has been performed in rodents, but there is also evidence from clinical trials (Figure 4). Most notably, this includes drugs in the families of RAS inhibitors, mTOR inhibitors, statins, SGLT2 inhibitors, and vitamin D [5,7,8,255,256]. In humans, these agents generally increased circulating Klotho levels by 5 to 25%. Almost all clinical data are based on serum or plasma s-Klotho levels and do not necessarily apply to levels in the brain and other tissues. Clinical investigations in this area have been few and limited, and more extensive studies are warranted.

Some of these drug stimulatory effects are consistent with regulatory elements of the Klotho promoter (e.g., vitamin D response elements) as well as other regulatory factors. Because NF-κB binds to the Klotho promoter to suppress, it is suspected that anti-inflammatory agents will reverse this and increase Klotho, but evidence of this type is scant. Systemic lupus erythematosus (SLE) patients treated with prednisone had higher circulating Klotho [257]. It is unclear whether this was a direct effect of the drug. Related to this, the role of Klotho is not well defined in SLE, rheumatoid arthritis and systemic sclerosis [258].

Exercise of various type is effective at increasing Klotho, as reviewed by others [259,260,261]. It is not clear how exercise increases Klotho levels, but some mechanisms have been proposed [260]. Klotho production in skeletal muscle is thought to be low, but it might be increased by exercise [262]. Interestingly, some of the Klotho-enhancing effects might be due to a reduction in fat [263]. In terms of benefits, Klotho might increase muscle mass by inhibiting myostatin and TGF-β.

### 9.2. Non-Linear Klotho Responses

As related to therapy, it is unknown what Klotho levels will be beneficial. There is evidence, albeit limited, that some responses are not linear. Data from the National Health and Nutrition Examination Survey (NHANES) collected between 2007 and 2016 (in most studies) revealed non-linear associations of the Klotho serum level with all-cause mortality, systemic inflammation index, accelerated aging, metabolic syndrome, hyperlipidemia, frailty, chronic kidney disease and cancer [17,264,265,266,267,268,269,270,271]. In these studies, levels below 600 pg/mL were generally associated with increased morbidity and mortality. Moderate Klotho levels (especially in the range of 700–1000 pg/mL) were linked to optimal outcomes. However, higher levels often provided minimal or no improvement (L-shaped curves) [17] or even- lower benefit (U-shaped curves) [264,265,269,270,271]. These reports have limitations. In most cases, the subjects were in the range 40 to 79 years old. Klotho was measured at the beginning on residual frozen serum with a single commercial ELISA assay. Levels during the subsequent course are unknown. Furthermore, the accuracy of this ELISA methodology has been questioned by some investigators [1,272]. Therefore, further studies are required to confirm these findings.

In published studies of the relationship of Klotho levels to disease, a caveat is that the FGF23/Klotho ratio is often not examined or reported. This is relevant because FGF23 is a strong suppressor of Klotho expression, and high levels of FGF23 are pathogenic. Bi et al. [266] examined this ratio as related to diabetes (T2D) and atherosclerosis. Both FGF23 and the FGF23/Klotho ratio were linearly positively correlated with the occurrence of T2D as well as increased carotid intimal thickness and atherosclerosis in subjects with T2D. In contrast, Klotho levels were negatively correlated with these events.

## 10. Conclusions

Klotho exerts several protective functions against inflammation and aging. The renal functions have received considerable attention, especially as related to FGF23-dependent phosphate and vitamin D homeostasis. Hyperphosphatemia, as occurs in Klotho deficiency, appears to have a role in aging especially as related to CPP deposition in tissues [145]. Here, we focused on other aspects relevant to aging (inflammaging) and chronic diseases. In this respect, Klotho inhibits major pathways involved in acute and chronic inflammation. From several published studies, we present evidence that Klotho inhibits the activation of NF-κB and the NLRP3 inflammasome. These are key components of the inflammatory machinery, which are involved in numerous diseases. Some mechanisms of action are proposed, but they are not fully elucidated and require further investigation. The enhancement of SIRT1 expression might be an important factor. Relevant to aging, Klotho stimulates the production of antioxidative enzymes through Nrf2 and FOXOs. This neutralizes cell-damaging ROS. Klotho also mitigates endoplasmic reticulum stress and preserves mitochondrial functions by processes that are poorly understood.

Fibrosis is a major consequence of inflammation and can lead to organ failure. Klotho blocks TGF-β and Wnt, which are both heavily involved in promoting EMT, EndMT and fibrosis. These inhibitory activities are relevant to age-related diseases [273]. An excessive loss of muscle mass (sarcopenia) occurs in aging and many diseases. Klotho blocks mediators such as myostatin that depress muscle mass. Importantly, there is evidence of neuroprotection in both mice and humans. This could result from the inhibition of neuroinflammation, improvement of myelination, reduction in cellular stresses (such as ROS), prevention of neuronal cell death (apoptosis, pyroptosis or other), and other mechanisms.

The majority of this work has been performed in animal models, which are extremely useful but can never completely duplicate human physiology or pathology. In vitro studies have been performed with human cells or tissues (e.g., brain organoids). However, clinical trials have often been restricted to the analysis of circulating s-Klotho levels, which are generally depressed in aging and chronic diseases. There are several possibilities for human therapy, with either Klotho protein, peptides, Klotho-enhancing drugs, exercise or other approaches. Klotho does not appear to be toxic, but higher than normal levels (as seen in rare human diseases) can induce hypophosphatemia and other negative effects [274]. Furthermore, recent findings from NHANES studies suggest that moderate physiological Klotho levels are equal or superior to higher levels, at least concerning some pathologies. Thus, it might not be necessary to achieve supranormal Klotho levels for therapy. A relatively easy way to increase Klotho is through exercise. Interestingly, Klotho has been proposed as a biomarker for monitoring lifestyle improvement strategies [275], and this aspect deserves further investigation. In conclusion, Klotho is a multifunctional protein with a great potential for promoting the health, longevity and the treatment of several major diseases. The clinical aspects are manifestly underdeveloped, and considerable effort is necessary to produce and evaluate Klotho-based therapies.

## Figures and Tables

**Table 2:** Klotho (KL) effects on cognition and neurodegenerative diseases in humans and primates.

Species/Disease	Sample	Techniques	Main Findings	Reference
Human/AD	Serum and CSF	ELISA	KL serum and CSF levels positively correlated with cognition, negatively correlated with dementia rating (apoE4 positive or not).	[224]
Human	Serum	ELISA	KL serum levels positively correlated with cognition (60–79 year old subjects).	[226]
Human/AD	Serum and CSF	ELISA	KL-VS variant carriers had higher KL levels than noncarriers.	[222]
Human/AD	Brain (in vivo)	PET	KL-VS^het^ associated with lower tau, better memory.	[220]
Human/AD	CSF and plasma	ELISA	KL lower in CSF of AD; high KL in CSF associated with improved tau and Aβ biomarkers in CSF.	[223]
Human/AD & FTD	Blood mononuclear cells	qPCR	KL was lower in cells from AD and FTD; no KL-VS effect.	[228]
Human/cerebrovascular disease (stroke)	Plasma	ELISA, cerebral imaging	Higher KL levels associated with lesser cerebral small vessel disease.	[217]
Human	Brain organoids	In vitro culture	KL inhibited neuronal senescence.	[221]
Human	Neural hippocampal progenitors	In vitro culture	KL increased neuronal differentiation and reduced cell death by apoptosis.	[218]
Nonhuman	Serum	KL s.c., cognitive	KL injection improved performance in cognitive tests.	[225]
Human	N/A	N/A	KL functions in the brain.	[216,219,229]

**Abbreviations:** Aβ, amyloid-β; AD, Alzheimer’s disease; ApoE4, apolipoprotein E4; CSF, cerebrospinal fluid; ELISA, enzyme-linked immunosorbent assay; FTD, frontotemporal dementia; het, heterozygous; KL, α-Klotho; N/A, not applicable; PET, positron emission tomography; qPCR, quantitative polymerase chain reaction; s.c., subcutaneous injection.

**Table 3:** Evidence that Klotho (KL) is neuroprotective in experimental models of disease.

Disease Model	Methods	Main Findings	Reference
Klotho hypomorphic mouse (*Kl^kl/kl^*)	Phenotypic analysis	Markedly impaired cognition; ↑ oxidative stress; ↑ neuronal cell death in hippocampus. Countered by an antioxidant.	[60]
Klotho knockout mouse (*Kl*^−/−^)	RNA species brain analysis	Altered expression of long RNAs, microRNAs and tRFs similar to AD.	[252]
AD (APP/PS1 mouse)	*KL* lentivirus vector (i.c.v.)	KL overexpression: Improved cognition; ↑ autophagy; ↓ Aβ; ↓ CAA; ↓ NLRP3 inflammasome activation.	[80,250]
AD (human APP-J20 mutant mouse)	KL overexpression (transgenic)	KL reduced premature mortality and loss of NMDA receptors in hippocampus; ↑ synaptic function and memory.	[249]
Knockout of *KL* in choroid plexus (CP), mouse	*KL* knockout in vivo; mϕ + LPS + KL in vitro	KL knockout: ↑ inflammatory mediators and ↑ mϕ in CP; ↑ microglial activation in hippocampus. KL in vitro (mϕ): ↓ TXNIP and ↓ NLRP3 inflammasome activation.	[109]
Mouse (aging)	KL s.c. injection.	KL (or KL1 domain only): ↑ cognition and ↑ synaptic plasticity in hippocampus.	[251]
Mouse (postnatal or embryo)	Cultures of neurons or astrocytes	In astrocytes, KL ↑ FOXO3a and protected against oxidative stress. In neurons, KL ↑ proteasomal activity.	[253]
Rat temporal lobe epilepsy (TLE)	Induced TLE. KL vector injected into hippocampus	KL countered cognitive deficit, and was neuroprotective. It suppressed ROS. It prevented cell death by ferroptosis.	[115]
Rat cells and KL deficient mice	In vitro culture, brain tissue analysis	KL enhanced oligodendrocyte maturation and myelination, in vitro and in vivo.	[248]
Hippocampal neurons	Multiple KL actions	KL protects by regulating ROS, DNA damage, inflammation, autophagy, ER stress and cell death.	[131]

**Abbreviations:** ↓, decreased; ↑, increased; +, added to the culture; Aβ, amyloid-β; AD, Alzheimer’s disease; APP, amyloid precursor protein; APP/PS1, amyloid precursor protein/presenilin-1; CAA, cerebral amyloid angiopathy; CP, choroid plexus; CSF, cerebrospinal fluid; ER, endoplasmic reticulum; i.c.v., intracerebroventricular; KL, α-Klotho; LPS, lipopolysaccharide; mϕ, macrophage; p-tau, phosphorylated tau protein; qPCR, quantitative polymerase chain reaction; s.c., subcutaneous injection; TLE, temporal lobe epilepsy; tRFs, tRNA fragments; TXNIP, thioredoxin-interacting protein.
